# Unraveling the role of *LDHA* and *VEGFA* in oxidative stress: A pathway to therapeutic interventions in cerebral aneurysms

**DOI:** 10.17305/bb.2024.10510

**Published:** 2024-06-01

**Authors:** Jiaying Wu, Lixia Lu, Beibei Dai, Aiyong Yu

**Affiliations:** 1 Department of Neurology, Songjiang Hospital Affiliated to Shanghai Jiao Tong University School of Medicine, Shanghai, China; 2 Department of Neurology, Qingpu Branch of Zhongshan Hospital, Fudan University, Shanghai, China; 3 Department of Ultrasound, Obstetrics and Gynecology Hospital of Fudan University, Shanghai, China

**Keywords:** Cerebral aneurysms, lactate dehydrogenase A (LDHA), glycolysis, lactate metabolism, pathogenesis, therapeutic targets, blood vessels, enzyme, molecular mechanisms

## Abstract

Cerebral aneurysms (CAs) are critical conditions often associated with oxidative stress in vascular endothelial cells (VECs). The enzyme lactate dehydrogenase A (LDHA) plays a crucial role in glycolysis and lactate metabolism, processes implicated in the pathogenesis of aneurysms. Understanding these molecular mechanisms can inform the development of novel therapeutic targets. This study investigated the role of lactate metabolism and lactate-related genes, particularly *LDHA* and vascular endothelial growth factor A (*VEGFA*) genes, in VECs during oxidative stress. Using the GSE26969 dataset, we identified the differential expression of lactate-related genes and performed functional enrichment analysis, revealing significant associations with glycolysis and lactate metabolic pathways. To induce oxidative stress, VECs were treated with H_2_O_2_, and the expression of *LDHA* and *VEGFA* was analyzed using quantitative real-time polymerase chain reaction (qRT-PCR) and western blotting (WB) assays. Under oxygen-glucose deprivation/reperfusion (OGD/R) conditions, the effects of *LDHA* overexpression and *VEGFA* knockdown on cell viability and apoptosis were evaluated. Immunoprecipitation (IP) combined with WB was used to detect the lactylation status of *LDHA* following OGD/R stimulation and treatment with lactic acid (LA) and 2-deoxyglucose (2-DG). Our results indicated that oxidative stress modulates *LDHA* expression, glucose uptake, and lactate production, suggesting a metabolic shift toward glycolysis. *LDHA* overexpression improved cell survival and reduced apoptosis, while *VEGFA* knockdown had the opposite effect. Additionally, 2-DG treatment reduced *LDHA* lactylation and apoptosis. Our findings demonstrated that LDHA plays a critical role in the oxidative stress response of VECs, highlighting the potential therapeutic value of targeting glycolysis in CA. This study contributes to the understanding of metabolic adaptations in vascular pathologies and suggests new avenues for therapeutic intervention in CA management.

## Introduction

Cerebral aneurysms (CAs), also known as intracranial aneurysms (IA), are defined by an abnormal dilation of the cerebral arteries [1]. This vascular disease is characterized by isolated and weakened segments of the artery wall, which are prone to rupture and can have potentially fatal consequences [2]. CAs are the leading cause of spontaneous subarachnoid hemorrhage (SAH), ranking third among cerebrovascular accidents behind cerebral thrombosis and hypertensive cerebral hemorrhage [3]. Its high mortality and prevalence in cardiovascular diseases have garnered significant attention. In Central and Eastern European countries, the estimated incidence of CA among adults is 3.2% [4, 5]. The multifaceted pathogenesis of CA involves various factors, including genetic predisposition, hemodynamic stress, and environmental influences [6]. The mechanisms of ruptured IA involve several biological processes (BPs), such as inflammation, phenotypic changes in vascular smooth muscle cells (VSMCs), cell adhesion, atherosclerosis, and abnormalities in extracellular matrix (ECM) metabolism [7]. Current evidence suggests that abnormal inflammatory responses in the vessel wall triggered by hemodynamic changes induce the activation of signaling pathways, such as the nuclear factor kappa-light-chain-enhancer of activated B cells (NF-κB) pathway, through unknown mechanisms. This process leads to the production of matrix metalloproteinases (MMPs), such as MMP2 and MMP9, which in turn trigger ECM degradation and phenotypic changes in VSMCs, ultimately leading to aneurysm formation and rupture [8]. Inflammatory smooth muscle cells (iSMCs) have been reported to induce endothelial cell (EC) dysfunction and drive the progression of IA. Phoenixin-14 (PNX-14) is a newly identified brain peptide with pleiotropic effects, involved in the regulation of reproduction, cardioprotection, lipid deposition, and glucose metabolism. Previous studies have shown that PNX-14 protects brain ECs from oxygen-glucose deprivation/reoxygenation (OGD/R)-induced cellular damage [9]. Currently, endovascular treatments offer a viable alternative, circumventing some side effects associated with open surgery while yielding favorable outcomes [10]. Despite significant strides in comprehending molecular mechanisms, diagnostic modalities, and treatment strategies, further exploration is imperative to enhance our understanding of CA.

Lactic acid (LA), an organic acid produced through glycolysis, plays a key role in cellular energy metabolism, particularly under hypoxic conditions, where glucose is metabolized into lactate, yielding limited ATP [11]. The process of LA fermentation has garnered considerable attention, with researchers actively exploring its implications in various diseases, including tumors and cancers. Isoglycyrrhizin (ISL) significantly inhibited gastric cancer (GC) growth and increased apoptosis. ISL regulated the expression of proteins related to apoptosis and metabolism both in vivo and in vitro. ISL inhibited mitochondrial oxidative phosphorylation (OXPHOS) and glycolysis by blocking glucose uptake and inhibiting lactate production and secretion, which was accompanied by increased accumulation of reactive oxygen species (ROS) [12]. Low dedicator of cytokinesis 8 (DOCK8) protein expression induced extracellular acidification rate (ECAR) and promoted hexokinase 2 (HK-2), pyruvate kinase M2 (PKM2), and lactate dehydrogenase A (LDHA) protein expression, which in turn increased pyruvate, lactate, and ATP content. In contrast, treatment with 2-deoxy-D-glucose (2-DG) was able to reverse these effects. These results suggest that DOCK8 may suppress sepsis-induced neutrophil immune function by regulating aerobic glycolysis and causing excessive inflammation [13]. Cardamonin further increased intracellular ROS levels by inhibiting the nuclear factor erythroid 2-related factor 2 (Nrf2)-dependent ROS scavenging system. The accumulation of ROS eventually induced apoptosis in breast cancer cells. Additionally, cardamonin treatment reduced glucose uptake as well as lactate production and efflux, indicating its important role in inhibiting glycolysis [14]. Gao et al. [15] revealed that enhanced lactylation at the fatty acid synthase K673 site is a downstream mechanism of decreased liver lipid accumulation following mitochondrial pyruvate carrier 1 (MPC1) downregulation in nonalcoholic fatty liver disease (NAFLD). Gu et al. [16] discovered the significance of lactate as a crucial tumor metabolite, demonstrating that lactylation of membrane-organizing extension spike protein (MOESIN) enhances the stability and function of regulatory T (Treg) cells. This lactylation, in turn, promotes transforming growth factor-beta (TGF-β) signaling, facilitating tumorigenesis and suggesting potential therapeutic strategies for anti-tumor immunity. In a study by Yang et al. [17], the inactivation of von Hippel–Lindau (VHL) in clear cell renal cell carcinoma (ccRCC) was found to trigger histone lactylation, establishing a positive feedback loop with platelet-derived growth factor receptor beta (PDGFRβ) signaling that promotes ccRCC progression. Correction of aberrant histone lactylation effectively inhibits ccRCC growth, and combined inhibition with PDGFRβ presents a promising therapeutic approach. Therefore, given the compelling research on lactylation in diverse diseases, the relationship between lactylation and IA warrants comprehensive exploration in our ongoing studies.

Cellular metabolism maintains normal cellular function by meeting its energy and metabolite requirements [18]. Human lactate dehydrogenase (LDH), which consists of two subunits, LDHA and lactate dehydrogenase B (LDHB), acts as a key glycolytic enzyme catalyzing the interconversion of pyruvate and lactate in the anaerobic glycolytic pathway [19]. Although the role of lactate LDHB as a terminal metabolizing enzyme in the glycolytic pathway has been extensively studied in cancer cells, relatively little research has focused on LDHA [20]. As a component of the LDH family, LDHA is predominantly expressed in muscle tissues [21]. Its potential role in providing lactate molecules for lactylation modifications, influencing various cellular processes, has sparked growing interest in understanding the broader cellular functions regulated by lactate [22]. In the context of osteogenesis, Nian et al. [23] observed increased glucose metabolism to lactate during osteoblast differentiation, with LDHA playing a crucial role. Reduced LDHA levels impair the formation of mineralized nodules and alkaline phosphatase (ALP) activity, indicating the importance of LDHA in osteogenesis. Chen et al. [24] demonstrated the crucial role of LDHA in promoting cardiomyocyte proliferation and enhancing cardiac repair post-myocardial infarction, linking it to succinyl coenzyme A reduction, thioredoxin reductase 1 (Txnrd1) ubiquitination inhibition, ROS alleviation, and M2 macrophage polarization. Similarly, Huo et al. [25] identified the signal transducer and activator of transcription 3 (STAT3)/long intergenic noncoding RNA 671 (LINC00671)/LDHA axis as a key regulatory pathway influencing glycolysis, growth, and lung metastasis in thyroid cancer. Abruzzo et al. [26] found in animal models that LDHA plays a key role in blood flow-induced dilation and remodeling of cerebral arteries, resulting in differences in susceptibility to cerebrovascular lesions among different genetic backgrounds. Knockdown of LDHA inhibited OGD/R-induced N2a cell scotomies, while the overexpression of high-mobility group box 1 (HMGB1) reversed this inhibition. Mechanistically, LDHA induces cellular focal death by targeting HMGB1 in cerebral ischemia/reperfusion (CI/R) injury and mediating histone lactylation [27]. Expanding on this understanding, our study focused on investigating the impact of LDHA modulation on vascular EC (VEC) proliferation and migration in brain aneurysm patients. By examining the effects of LDHA knockdown and overexpression on these cellular processes, we observed a promotive role of LDHA in cell proliferation and migration.

We sought to understand the molecular mechanisms of lactate metabolism in VECs under oxidative stress, with a particular emphasis on the involvement of LDHA and vascular endothelial growth factor A (VEGFA) in the pathogenesis of CA. Through a comprehensive analysis using the GSE26969 dataset, we identified key lactate-related genes and their enriched pathways related to CA. This study focused on *LDHA* as the hub gene and analyzed its key role in regulating the oxidative stress response of VECs and its possible influence on CA progression. Furthermore, we explored the interaction between LDHA and VEGFA, two key factors in cellular stress responses and vascular pathology. Our research emphasizes the significance of lactate metabolism and its regulation in cerebrovascular disorders, aiming to illuminate metabolic alterations in VECs during oxidative stress and identify potential treatment targets for CA.

## Materials and methods

The aim of the present study was to investigate the roles of LDHA and VEGFA in oxidative stress, as a potential pathway for therapeutic intervention in CA. To achieve this, we performed bioinformatics analyses and designed the following series of experiments.

### Download of IAs-related GSE26969 dataset and screening of differentially expressed genes (DEGs)

We retrieved the GSE26969 dataset from the Gene Expression Omnibus (GEO) (https://www.ncbi.nlm.nih.gov/gds/) database [28], which included three IA samples and three normal temporal artery samples. Differential expression analysis was performed using the limma package of R software [29]. Genes with a fold change (FC) > 2 were classified as up-regulated, whereas genes with an FC < 0.5 were considered downregulated, using a statistical significance threshold of *P* < 0.05.

### Acquisition and enrichment analysis of lactate-related genes in GSE26969 dataset

The Gene Set Enrichment Analysis (GSEA) (https://www.gsea-msigdb.org/gsea/index.jsp) website provides software tools and information to assist researchers in conducting gene set enrichment analysis, analyzing genomic data, and understanding gene expression patterns in various conditions and disorders. In this study, we downloaded 348 lactate-related genes from this database. Intersection analysis of 348 lactate-related genes, upregulated DEGs, and downregulated DEGs from the GSE26969 dataset was performed using the bioinformatics platform (https://bioinformatics.psb.ugent.be/webtools/Venn/) to obtain the overlapping genes. Functional enrichment analysis was then conducted using the Database for Annotation, Visualization, and Integrated Discovery (DAVID) (https://david.ncifcrf.gov/tools) tools. This included Kyoto Encyclopedia of Genes and Genomes (KEGG) pathway enrichment [30], and Gene Ontology (GO) term analysis [31], covering BPs, cellular components (CC), and molecular functions (MF). Results with *P* < 0.05 were considered statistically significant.

### Construction of protein–protein interaction (PPI) network and expression analysis of key overlapping genes

Following the identification of overlapping genes, we shifted our focus to the proteins encoded by these 63 genes. PPI networks were constructed using the Cytoscape software [32]. The Cytohubba plugin within Cytoscape was then employed, utilizing the Maximum Clique Centrality (MCC), Maximum Neighborhood Component (MNC), and EcCentricity algorithms to identify three key network modules. The bioinformatics platform (https://bioinformatics.psb.ugent.be/webtools/Venn/) was used again to perform intersection analysis on the genes in these three network modules, thereby obtaining the key overlapping genes. Finally, we analyzed the expression profiles of overlapping genes derived from the PPI network in the IA and control groups within the GSE26969 dataset, leading to the identification of the hub gene.

### Cell culture and oxidative stress induction

Primary VECs were provided by the Shanghai Institute of Biological Sciences (Shanghai, China). The VECs were cultured in Dulbecco’s Modified Eagle Medium (DMEM) (Gibco, USA) supplemented with 10% fetal bovine serum (FBS) and 1% penicillin–streptomycin (Gibco, USA) in a humidified atmosphere containing 5% CO_2_. Previous studies have shown that oxidative stress is a key factor in the formation and rupture of IAs. To simulate oxidative stress, VECs were exposed to 0.5 mM hydrogen peroxide (H_2_O_2_) for 12 h. VECs without added H_2_O_2_ served as the control group. The concentration and duration of H_2_O_2_ treatment were optimized in preliminary experiments to induce moderate levels of oxidative damage without causing excessive cell death [33].

### Cell treatment

Subsequently, these H_2_O_2_-preconditioned VECs were subjected OGD/R to simulate ischemia-reperfusion injury in vitro. This involved incubating cells in glucose-free DMEM under hypoxic conditions (1% O_2_) for 3, 6, 12, and 24 h, followed by reoxygenation with complete DMEM under normoxic conditions for 24 h. Under OGD/R stimulation, VECs were simultaneously treated with 25 mM LA to study the effects of acidic stress. Additionally, a separate set of VECs were treated with 2-DG at a concentration of 25 mM during the reoxygenation phase to evaluate the effect of glycolysis inhibition after OGD. Both treatments were maintained for 24 h post-OGD/R induction to elucidate the cellular response to ischemic stress and the therapeutic potential of metabolic modulators in the context of EC recovery and survival [34].

### Cell transfection

VECs were plated at a density of 2 × 10^5^ cells per well in 24-well plates to achieve optimal confluence for transfection. To regulate the *LDHA* expression, VECs were transiently transfected with plasmids designed for *LDHA* overexpression (over-LDHA) and three different small interfering RNAs (siRNAs) targeting *LDHA* (si-LDHA-1, si-LDHA-2, si-LDHA-3) for gene knockdown. Similarly, a *VEGFA*-specific overexpression plasmid (over-VEGFA) was used to overexpress *VEGFA*, while siRNA targeting *VEGFA* was used to knock it down. Transfections were conducted using Lipofectamine 3000 reagent (Invitrogen, USA) following the manufacturer’s instructions.

### Quantitative real-time polymerase chain reaction (qRT-PCR) assay

Total RNA was extracted from VECs using the TRIzol reagent (Thermo Fisher Scientific, USA) as per the manufacturer’s guidelines. The PrimeScript RT Reagent Kit (Takara, Japan) was used for cDNA synthesis. Subsequent qRT-PCR assays were performed using the SYBR Green PCR Master Mix (Applied Biosystems, USA) on a StepOnePlus Real-Time PCR System (Applied Biosystems, USA). Gene expression was normalized to the internal standard, glyceraldehyde-3-phosphate dehydrogenase (GAPDH) [35]. The primer sequences used for amplification were as follows: *LDHA* forward: 5′-ATGGCAACTCTAAGGATCA-3′, *LDHA* reverse: 5′-GCAACTTGCAGTTCGGGC-3′; *VEGFA* forward: 5′-CGAAAGCGCAAGAAAT-3′, *VEGFA* reverse: 5′-CTCCAGGGCATTAGACAGC-3’. For the reference gene *GAPDH*, the primers were: *GAPDH* forward: 5′-CAAGCTCATTTCCTGGTATGAC-3′, *GAPDH* reverse: 5′-CAGTGAGGGTCTCTCTCTTCCT-3′. Expression analysis utilized the 2^-ΔΔCT^ method with GAPDH as the internal control.

### Western blotting (WB) assay

Protein lysates obtained from VECs were prepared using the radioimmunoprecipitation assay (RIPA) lysis buffer (Thermo Fisher Scientific, USA) supplemented with protease and phosphatase inhibitors (Thermo Fisher Scientific, USA). The BCA Protein Assay Kit (Thermo Fisher Scientific, USA) was employed to quantify protein concentrations. Equal amounts of protein were separated by the sodium dodecyl sulfate-polyacrylamide gel electrophoresis (SDS-PAGE) and subsequently transferred to the polyvinylidene difluoride (PVDF) membranes (Millipore, USA). The membranes were probed with primary antibodies targeting LDHA (1:100; Cell Signaling Technology, Boston, USA), VEGFA (1:100; ABclonal, Wuhan, China), and GAPDH (1:5000, Cell Signaling Technology) as a loading control. After incubation with secondary antibodies, bands were visualized using enhanced chemiluminescence (ECL) and captured using a ChemiDoc system.

### Assessing cell viability using the cell counting kit-8 (CCK-8) assay

For the cell viability assay, VECs were seeded at a density of 5 × 10^3^ cells per well in a 96-well plate. After the specified treatments, 10 µL of CCK-8 solution was added to each well, and the cells were incubated for 1–4 h at 37 ^∘^C. The optical density was measured at 450 nm using a microplate reader, with higher absorbance indicating greater cell viability. Each experimental setup was replicated three times to ensure consistency and statistical reliability.

### Flow cytometry

For flow cytometric analysis, VECs were detached using trypsin-ethylenediaminetetraacetic acid (EDTA) (Gibco, USA), followed by a PBS wash. Cells were then stained using an Annexin V-fluorescein isothiocyanate (FITC)/propidium iodide (PI) apoptosis detection kit, according to the manufacturer’s instructions. Flow cytometry was performed on a flow cytometer (BD Biosciences, USA), and data analysis was conducted using FlowJo software (FlowJo LLC, USA).

### Migration assay

Twenty-four hours post-transfection, cells were harvested and resuspended at a concentration of 5 × 10^4^ cells/well. These cells were added to the upper chamber of a Transwell insert (6-well format), while the lower chamber contained complete medium as a chemoattractant. After 48 h of incubation at 37 ^∘^C, nonmigratory or noninvasive cells remaining in the upper chamber were gently removed with a cotton swab. The cells on the underside of the membrane, representing migrated or invaded cells, were fixed with 4% paraformaldehyde, stained with DAPI for nucleus visualization, and counted under a fluorescence microscope. Captured images were documented for further analysis [36].

### Measurement of lactate production and ECAR

VECs were treated with 0.5 mM H_2_O_2_ under OGD/R conditions. Glucose uptake and lactate production were assessed using a glucose uptake assay kit (Rsbio, Shanghai, China) and a lactate assay kit (Biotime, Shanghai, China). Concurrently, the pH of the cell medium was measured to determine changes due to metabolic activity. ECAR was measured using a Seahorse XF Analyzer (Agilent Technologies, Beijing) to track metabolic changes in VECs after H_2_O_2_ and OGD/R therapy. VECs were sequentially exposed to glucose, oligomycin A, and 2-DG to assess glycolytic capacity and glycolytic reserve. Data were captured in real time and plotted as ECAR against time to illustrate the cellular metabolic response to the treatments [37].

### Immunoprecipitation (IP) and WB analysis of lactate-LDHA

Cells were lysed using ice-cold RIPA buffer supplemented with protease and phosphatase inhibitors. For IP, VECs treated with 0.5 mM H_2_O_2_ were lysed, and LDHA was isolated using an anti-LDHA antibody and Protein A/G beads. After overnight incubation, the complexes were washed and eluted for SDS-PAGE and western blot analysis. Blots were probed with anti-lactoyllysine to detect lactylation modifications and with anti-LDHA to assess total LDHA levels. To assess dynamic lactylation after H_2_O_2_ treatment, VECs were incubated with 0.25 mM LA and subjected to IP at intervals of 0, 8, 16, and 24 h. Additionally, cells were treated with 2-deoxyglucose (2-DG) after OGD/R to examine the effect of glycolysis inhibition on LDHA lactylation [38].

### Statistical analysis

Data analysis was performed using the R programming language. Intergroup differences were assessed using Student’s *t*-test, with all data presented as mean ± SD. For comparisons across multiple groups, Tukey’s post-hoc test, in conjunction with analysis of variance (ANOVA), was applied. Statistical significance was defined as *P* < 0.05 [39].

**Figure 1.**
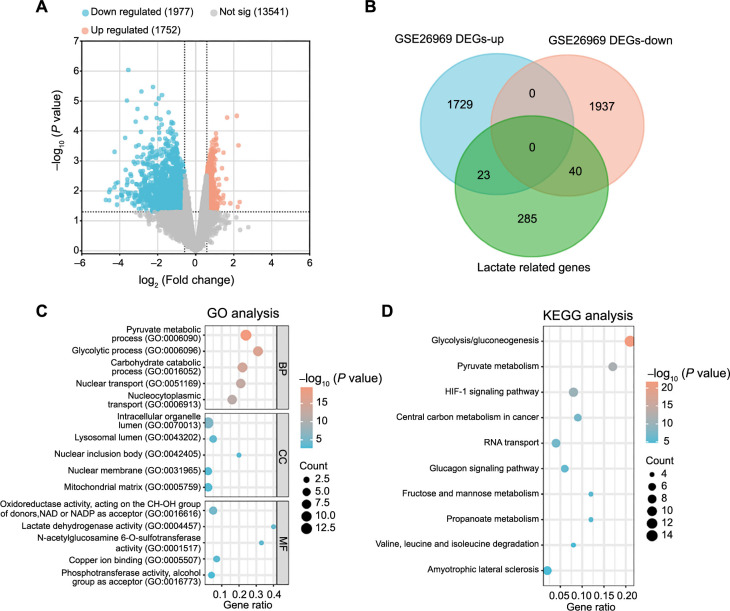
**Comprehensive bioinformatics analysis of lactate-related gene expression.** (A) The volcano plot visually representing the DEGs identified from the GSE26969 dataset. The *x*-axis (log_2_ fold change) signifies the logarithmic FC in gene expression between tumor and normal samples. The *y*-axis (−log_10_ *P* value) denotes the statistical significance of the observed differential expression, with higher values indicating increased significance. Each point on the plot corresponds to an individual gene, with upregulated DEGs depicted in red and downregulated DEGs in blue. (B) The Venn diagram illustrating the intersection of lactate-related genes with both upregulated and downregulated DEGs, providing insights into genes associated with lactate metabolism. (C) GO enrichment analysis showcasing the functional roles of the overlapping genes, categorizing them into BP, CC, and MF. The abscissa represents GeneRatio, and the ordinate depicts the enrichment terms. Larger dots indicate a higher number of enriched genes. (D) KEGG enrichment analysis predicting the pathways in which the overlapping genes are involved, offering a comprehensive view of the BPs associated with these genes. DEGs: Differentially expressed genes; GO: Gene Ontology; BP: Biological process; CC: Cellular component; MF: Molecular function; KEGG: Kyoto Encyclopedia of Genes and Genomes.

## Results

### Functional enrichment analysis of lactate-related genes in IA

Using the R package, we performed differential expression analysis on the GSE26969 dataset, comparing three IA samples with three normal temporal artery samples. This analysis identified a total of 1725 upregulated and 1977 downregulated DEGs (Figure 1A). Venn analysis, incorporating 348 lactate-related genes from the GSEA database alongside the upregulated and downregulated DEGs from the GSE26969 dataset, revealed 63 overlapping genes (Figure 1B). Functional enrichment analysis of these overlapping genes highlighted significant enrichment in various GO terms. In the category of BP, the genes were notably enriched in processes, such as glycolytic process, pyruvate metabolic process, and carbohydrate catabolic process. For CC terms, enrichment was observed in lysosomal lumen, mitochondrial matrix, and nuclear membrane. MF analysis indicated enrichment in LDH activity, oxidoreductase activity (acting on the CH–OH group of donors, nicotinamide adenine dinucleotide [NAD] or NAD phosphate [NADP] as acceptor), and phosphotransferase activity (alcohol group as acceptor), among others (Figure 1C). Furthermore, pathway analysis using the KEGG revealed significant enrichment in signaling pathways, such as glycolysis/gluconeogenesis, fructose and mannose metabolism, and pyruvate metabolism (Figure 1D).

### Identification of key genes in IA through PPI network analysis

**Figure 2.**
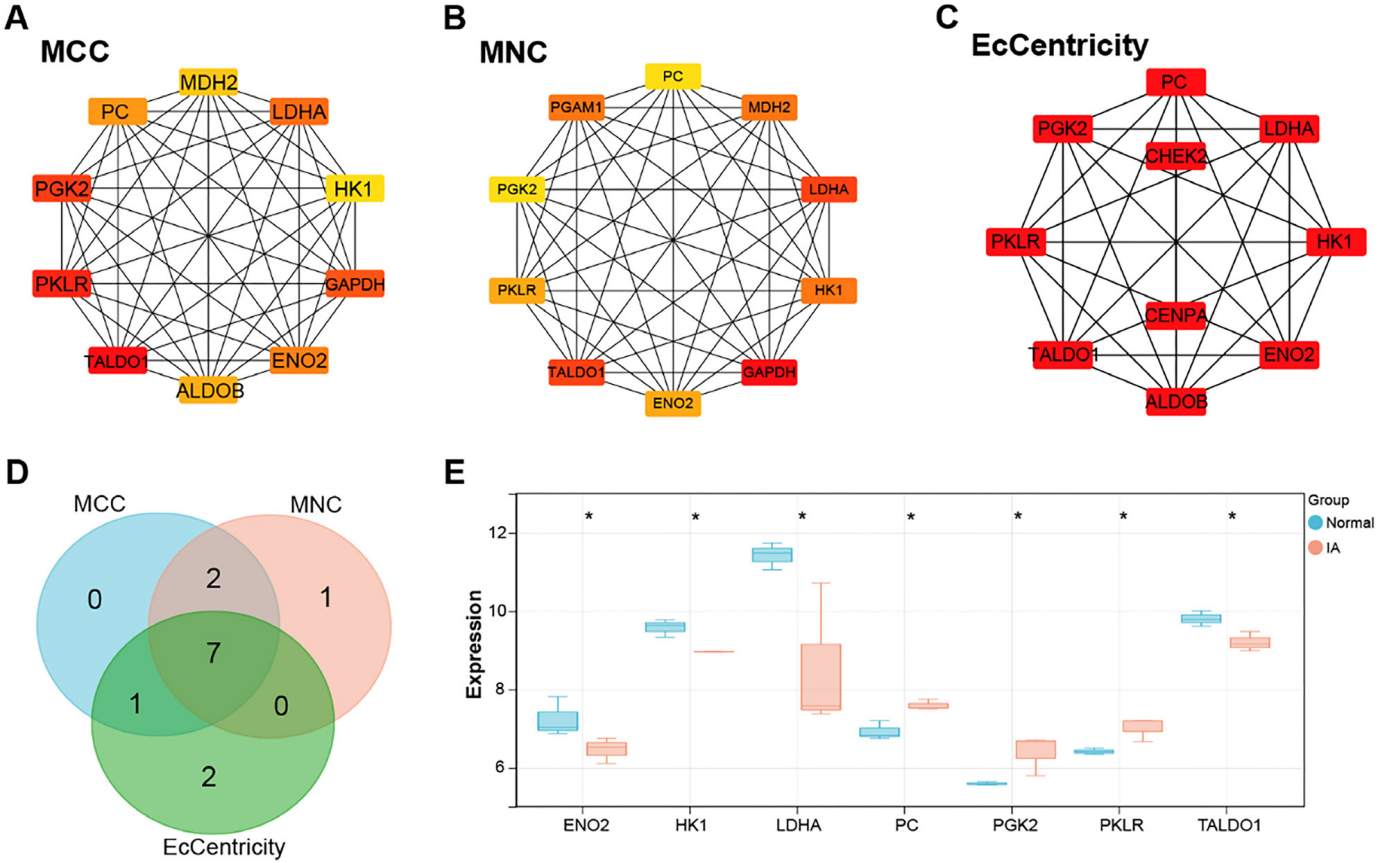
**Hub genes implicate *LDHA* in IA metabolic dysregulation.** (A–C) Analysis of PPI networks using MCC, MNC, and EcCentricity algorithms, revealing ten highly interconnected genes. The MCC network comprises ten nodes and 45 edges (A); the MNC network consists of ten nodes and 45 edges (B); and the EcCentricity algorithm-derived network includes ten nodes and 29 edges (C); (D) The Venn diagram illustrating the intersection of the ten highly interconnected genes identified by the MCC, MNC, and EcCentricity algorithms, highlighting seven common genes shared across all three methods; (E) Boxplots depicting the expression levels of overlapping genes (*ENO2, HK1, MDH2, ALDOB, LDHA, PKLR,* and *TALDO1*) in the IA and normal groups from the GSE26969 dataset. PPI: Protein–protein interaction; MCC: Maximal clique centrality; MNC: Maximum neighborhood component; LDHA: Lactate dehydrogenase A; IA: Intracranial aneurysms.

To further elucidate the relationships among the overlapping genes, we conducted a PPI network analysis. Employing three algorithms, MCC, MNC, and EPC, we identified ten highly interconnected genes, highlighting strong correlations within the network (Figure 2A–2C). Among these, a common set of seven genes (*ENO2, HK1, MDH2, ALDOB, LDHA, PKLR, TALDO1*) consistently emerged as significant. Subsequent analysis of the expression profiles of these genes in the IA and normal groups within the GSE26969 dataset revealed that *ENO2*, *HK1*, *LDHA*, and *TALDO1* were significantly downregulated in the IA group, whereas *PC*, *PGK2*, and *PKLR* were significantly upregulated (Figure 2D). Notably, our enrichment analysis unveiled the association of these genes predominantly with the lactate production process. Given that numerous studies have implicated lactate metabolism in the context of IA, we identified *LDHA*, a key gene involved in lactate production and cellular energy metabolism, as the hub gene for this study.

### Effects of *LDHA* regulation on VECs proliferation and migration under oxidative stress conditions

**Figure 3.**
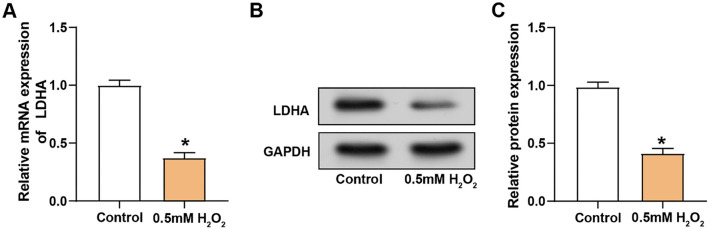
**Effect of oxidative stress on *LDHA* expression in VECs.** (A) Relative mRNA expression levels of LDHA in VECs under control conditions and after treatment with 0.5 mM H_2_O_2_; (B) WB analysis showing LDHA protein levels in VECs under control conditions and after treatment with 0.5 mM H_2_O_2_, using GAPDH as the loading control; (C) Quantification of LDHA protein expression normalized to GAPDH, demonstrating a significant decrease in protein levels after H_2_O_2_ exposure. *P < 0.05. LDHA: Lactate dehydrogenase A; VECs: Vascular endothelial cells; GAPDH: Glyceraldehyde-3-phosphate dehydrogenase; WB: Western blotting.

**Figure 4.**
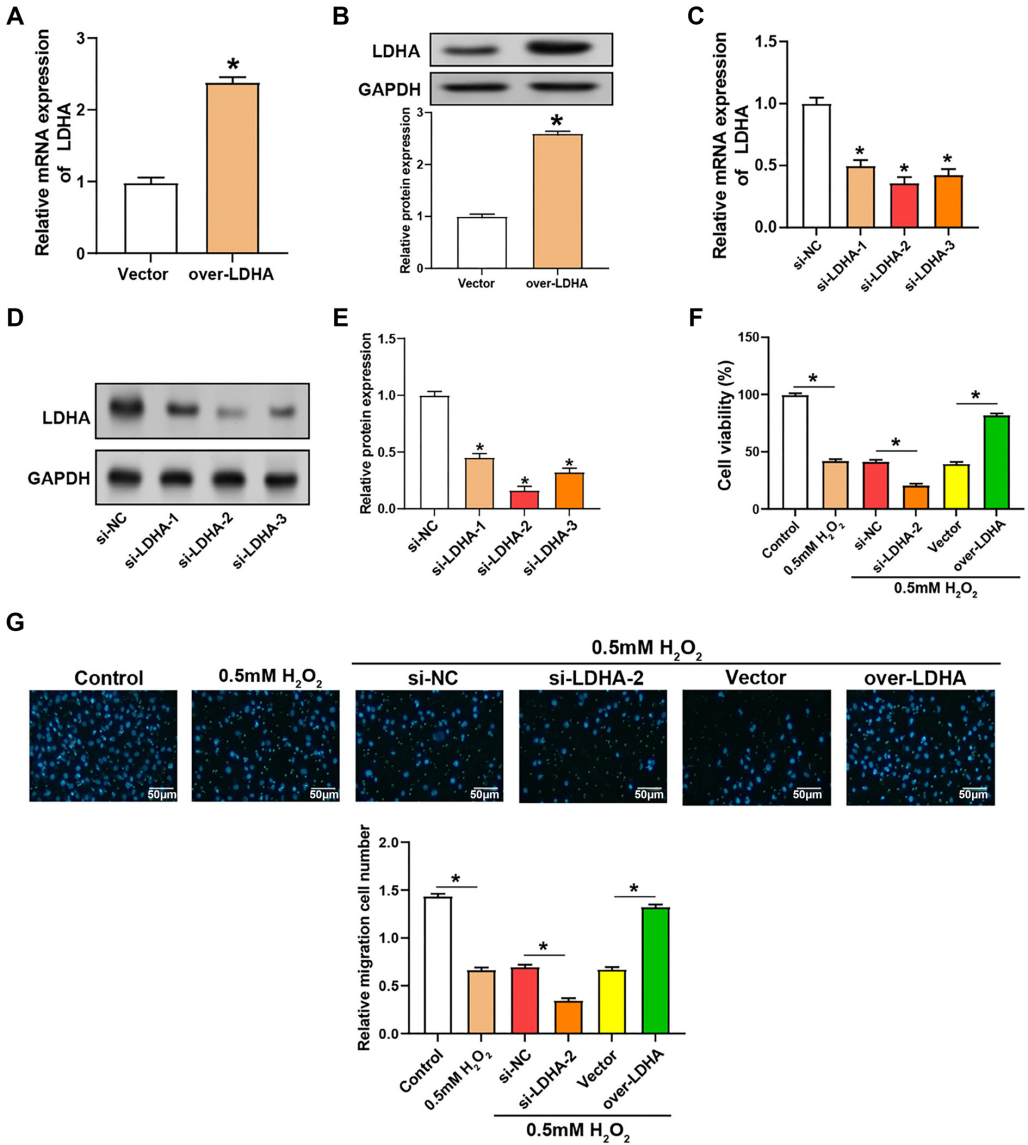
**Regulation of *LDHA* affects the viability of VECs induced by H_2_O_2_.** (A and B) qRT-PCR (A) and WB (B) analysis of relative mRNA expression and protein levels of LDHA in H_2_O_2_-treated VECs transfected with an overexpression vector (over-LDHA) or control vector; (C and D) LDHA expression levels in H_2_O_2_-treated VECs transfected with siRNA constructs targeting *LDHA* (si-*LDHA*-1, si-*LDHA*-2, si-*LDHA*-3) or a nontargeting control (si-NC); (E) CCK-8 assay measuring the viability of VECs treated with H_2_O_2_ and transfected with si-*LDHA*-2 or over-LDHA. The *y*-axis depicts the percentage of viable cells, and the *x*-axis shows various treatment conditions; (F) Transwell assay detecting the migration of VECs treated with H_2_O_2_ under different conditions (Control, 0.5 mM H_2_O_2_, si-NC, si-*LDHA*-2, Vector, over-LDHA). The six panels above represent migrating cells, and the bar graphs quantify the number of migrating cells under different treatment conditions; (G) The upper panel shows representative images of migrating cells in the Transwell assay under different treatment conditions. The lower panel displays a bar graph quantifying the relative migration cell number. *P* < 0.05. LDHA: Lactate dehydrogenase A; qRT-PCR: Quantitative real-time polymerase chain reaction; WB: Western blotting; VECs: Vascular endothelial cells.

We conducted a comprehensive analysis of LDHA expression using qRT-PCR and WB assays. Our results showed that LDHA mRNA expression was significantly reduced in VECs treated with H_2_O_2_ compared to the control group (Figure 3A). Consistent with mRNA expression, WB analysis also indicated a reduction in LDHA protein levels following H_2_O_2_ treatment (Figure 3B and 3C). We further evaluated the transfection efficiency of VECs transfected with either *LDHA* overexpression plasmid or *LDHA* knockdown plasmids (Figure 4A–4D). Among the knockdown plasmids, si-*LDHA*-2 exhibited the most significant knockdown efficiency and was thus selected for subsequent experiments. Functional assays using CCK-8 and Transwell showed significant changes in cell proliferation and migration. Specifically, knockdown of *LDHA* led to a significant decrease in both cell proliferation and migration, whereas overexpression of *LDHA* enhanced these cellular processes (Figure 4E and 4F).

### Downregulation of *VEGFA* inhibits the protective effect of overexpressed *LDHA* on H_2_O_2_-induced damage to VECs

**Figure 5.**
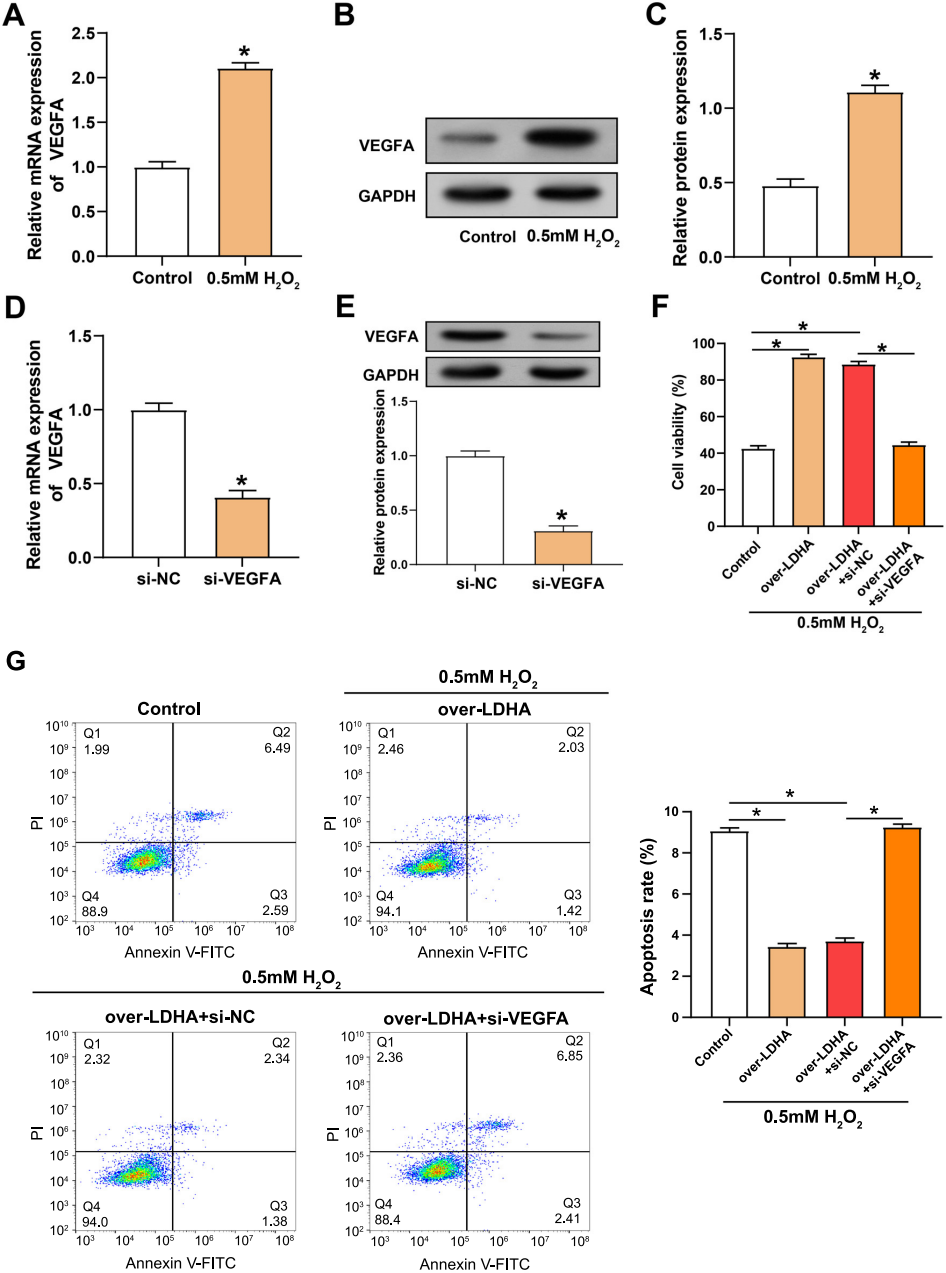
**Effects of *VEGFA* and *LDHA* expression on survival and apoptosis of H_2_O_2_-treated VECs.** (A) qRT-PCR detection of increased VEGFA mRNA levels in VECs after treatment with 0.5 mM H_2_O_2_; (B and C) WB analysis (B) and quantitative detection (C) showing VEGFA protein levels in VECs after 0.5 mM H_2_O_2_ exposure; (D and E) qRT-PCR (D) and WB (E) analysis of the transfection efficiency of si-VEGFA in VECs treated with 0.5 mM H_2_O_2_; (F) CCK-8 assay evaluating the effect of LDHA knockdown or overexpression on cell viability in VECs after 0.5 mM H_2_O_2_ treatment; (G) Flow cytometry apoptosis analysis showing the effect of LDHA knockdown or overexpression on the apoptotic rate of VECs under oxidative stress conditions. *P < 0.05. LDHA: Lactate dehydrogenase A; VEGFA: Vascular endothelial growth factor A; qRT-PCR: Quantitative real-time polymerase chain reaction; WB: Western blotting; CCK-8: Cell counting kit-8; VECs: Vascular endothelial cells.

Following treatment with 0.5 mM H_2_O_2_, VEGFA mRNA levels were significantly upregulated in VECs compared with untreated controls, indicating a cellular response to oxidative stress (Figure 5A). WB analysis corroborated these findings, showing an increase in VEGFA protein expression after H_2_O_2_ treatment (Figure 5B and 5C). To assess the role of VEGFA, we used siRNA to knock down VEGFA levels in VECs treated with 0.5 mM H_2_O_2_. Both qRT-PCR and WB detection confirmed a substantial decrease in VEGFA mRNA and protein levels, respectively, validating the efficiency of the gene silencing method (Figure 5D and 5E). Functional assays revealed that overexpression of LDHA increased cell viability, while knockdown of VEGFA negated this effect, underscoring the importance of VEGFA in cell survival under oxidative stress (Figure 5F). Flow cytometry analysis showed that cells overexpressing LDHA had reduced apoptosis, indicating a protective effect of LDHA. However, co-transfection with si-VEGFA and LDHA overexpression led to an increased apoptosis rate, suggesting that VEGFA knockdown may counteract the protective effects of LDHA overexpression (Figure 5G).

### Changes in LDHA expression, glucose uptake, lactate production, and glycolytic activity in VECs under OGD/R-induced oxidative stress

**Figure 6.**
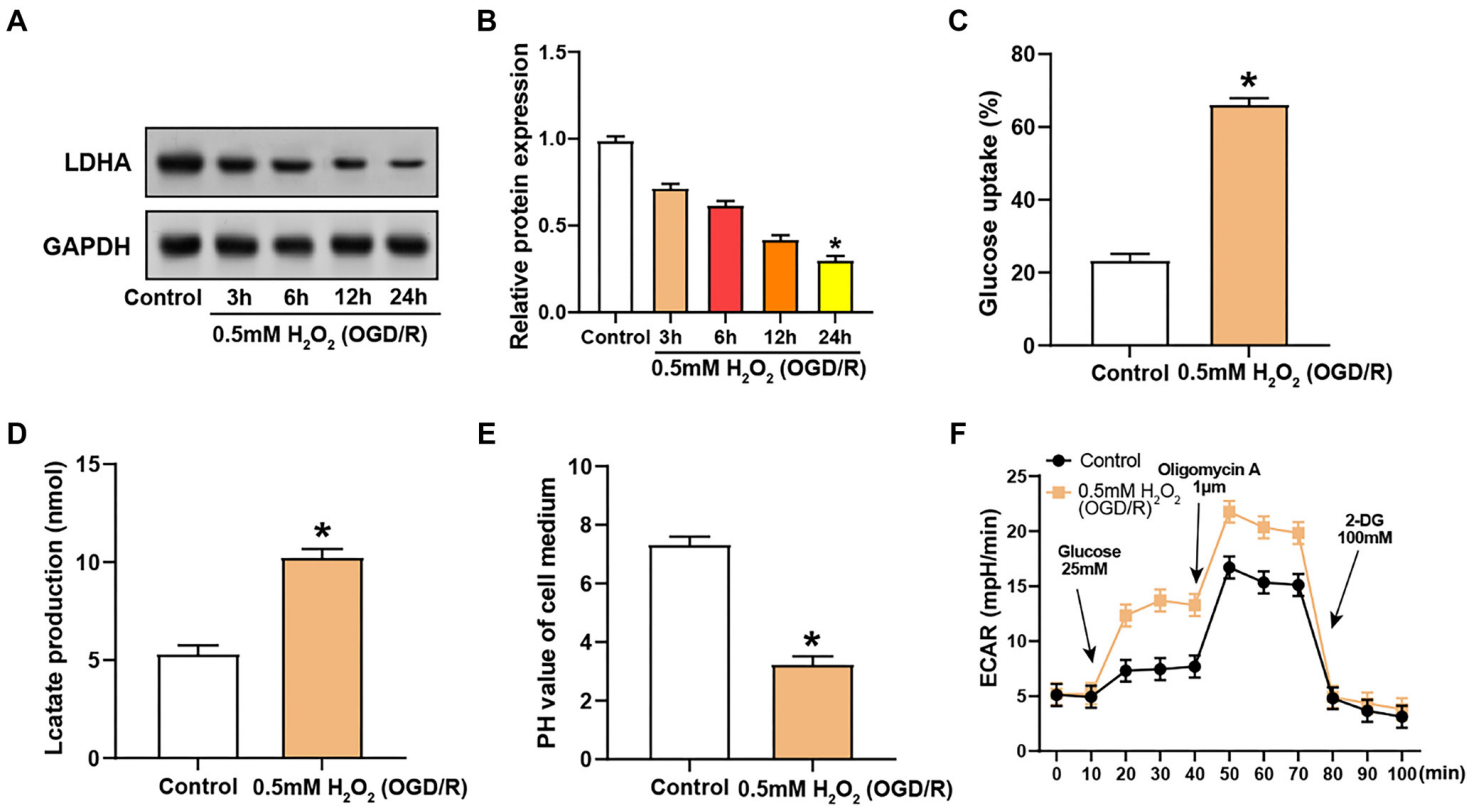
***LDHA* expression and glycolytic metabolism changes in VECs after OGD/R and H_2_O_2_ treatment.** (A and B) WB detection of LDHA protein levels in H_2_O_2_-treated VECs over time after OGD/R stimulation; (C) Glucose uptake levels in H_2_O_2_-treated VECs after OGD/R stimulation; (D) Lactate production levels in H_2_O_2_-treated VECs after OGD/R stimulation; (E) pH levels in the culture medium of H_2_O_2_-treated VECs after OGD/R stimulation; (F) ECAR values in H_2_O_2_-treated VECs after OGD/R stimulation; ECAR mapping shows results after glucose (25 mM), oligomycin A (1 µM), and 2-DG (100 mM) injection. *P < 0.05. LDHA: Lactate dehydrogenase A; WB: Western blotting; OGD/R: Oxygen-glucose deprivation/reperfusion; 2-DG: 2-deoxyglucose; ECAR: Extracellular acidification rate; VECs: Vascular endothelial cells.

WB analysis indicated that LDHA protein expression in VECs decreased in a time-dependent manner following exposure to 0.5 mM H_2_O_2_ during OGD/R, with a significant reduction observed 24 h after treatment (Figure 6A and 6B). This suggests that prolonged oxidative stress diminishes LDHA protein levels. The glucose uptake assay revealed a substantial increase in glucose absorption by VECs during OGD/R-induced oxidative stress, indicating increased glycolytic demand (Figure 6C). Correspondingly, treatment with 0.5 mM H_2_O_2_ under OGD/R conditions led to a significant increase in lactate production compared to controls, indicating a shift to anaerobic metabolism (Figure 6D). Additionally, the pH of the cell culture medium decreased sharply, indicating increased acidification due to elevated lactate production (Figure 6E). The ECAR curves showed dynamic metabolic adaptation following OGD/R induction, with notable responses after the addition of glucose and oligomycin A, and a considerable drop after the administration of 2-DG, demonstrating the VECs’ adaptation to oxidative stress (Figure 6F).

### *LDHA* overexpression and inhibition of glycolysis prevent the apoptosis of VECs cells under OGD/R-stimulated oxidative stress

**Figure 7.**
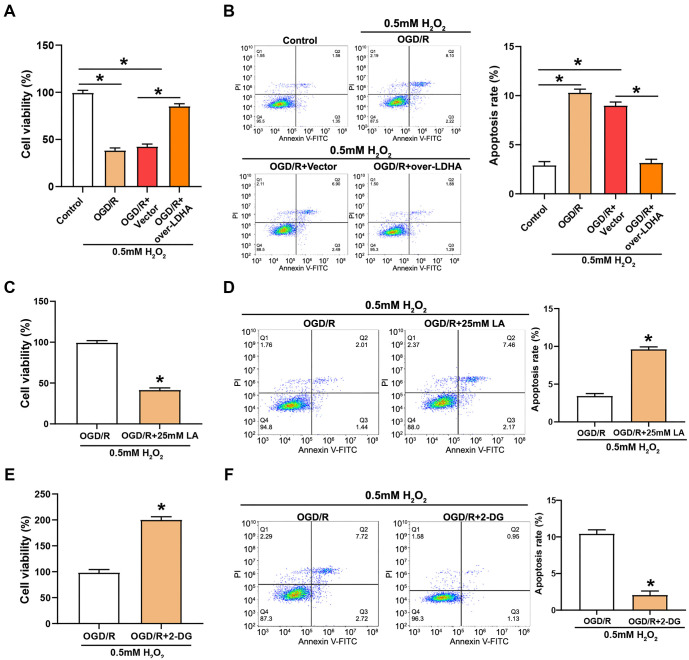
**Modulation of cellular proliferation and apoptosis in H_2_O_2_-treated VECs under OGD/R stress and *LDHA* overexpression.** (A) CCK-8 assay measuring cell viability of VECs treated with 0.5 mM H_2_O_2_ under OGD/R stimulation with/without *LDHA* overexpression. The *y*-axis represents the percentage of viable cells, and the *x*-axis represents different treatment conditions; (B) Flow cytometry analysis of the apoptosis rate in VECs treated with 0.5 mM H_2_O_2_ in different groups (OGD/R, OGD/R + Vector, OGD/R + over-LDHA). The *x*-axis represents fluorescence intensity, the *y*-axis represents cell count, and the bar graph illustrates the apoptotic rate; (C and D) CCK-8 assay (C) and flow cytometry (D) evaluating proliferation and apoptosis of H_2_O_2_-treated VECs under OGD/R treatment with the addition of 25 mM LA; (E and F) CCK-8 assay (E) and flow cytometry (F) assessing proliferation and apoptosis of H_2_O_2_-treated VECs under OGD/R treatment with the addition of 2-DG. *P < 0.05. LDHA: Lactate dehydrogenase A; OGD/R: Oxygen-glucose deprivation/reperfusion; LA: Lactic acid; 2-DG: 2-deoxyglucose; VECs: Vascular endothelial cells; CCK-8: Cell counting kit-8.

The cell viability assay showed a significant decrease in cell survival following OGD/R treatment compared to untreated controls. However, under the same stress conditions, cells overexpressing *LDHA* exhibited a significant increase in viability, indicating that *LDHA* provides protection against oxidative stress (Figure 7A). These findings were further supported by an examination of apoptosis. OGD/R markedly elevated apoptosis, and in cells overexpressing *LDHA*, apoptotic cell death was markedly enhanced (Figure 7B). Treatment of VECs with 25 mM LA following H_2_O_2_-induced oxidative stress and OGD/R led to a dramatical decrease in cell viability, indicating that excess lactate exacerbates damage caused by oxidative stress (Figure 7C). The modulatory role of lactate in stress-induced cell death was further highlighted by the increased apoptosis rate observed in the LA-treated group under OGD/R conditions (Figure 7D). In contrast, the administration of 2-DG after OGD/R stress in H_2_O_2_-treated VECs resulted in a significant increase in cell viability (Figure 7E) and a substantial reduction in the apoptosis rate (Figure 7F).

### Regulation of LDHA lactylation in VECs under OGD/R-induced oxidative stress condition

**Figure 8.**
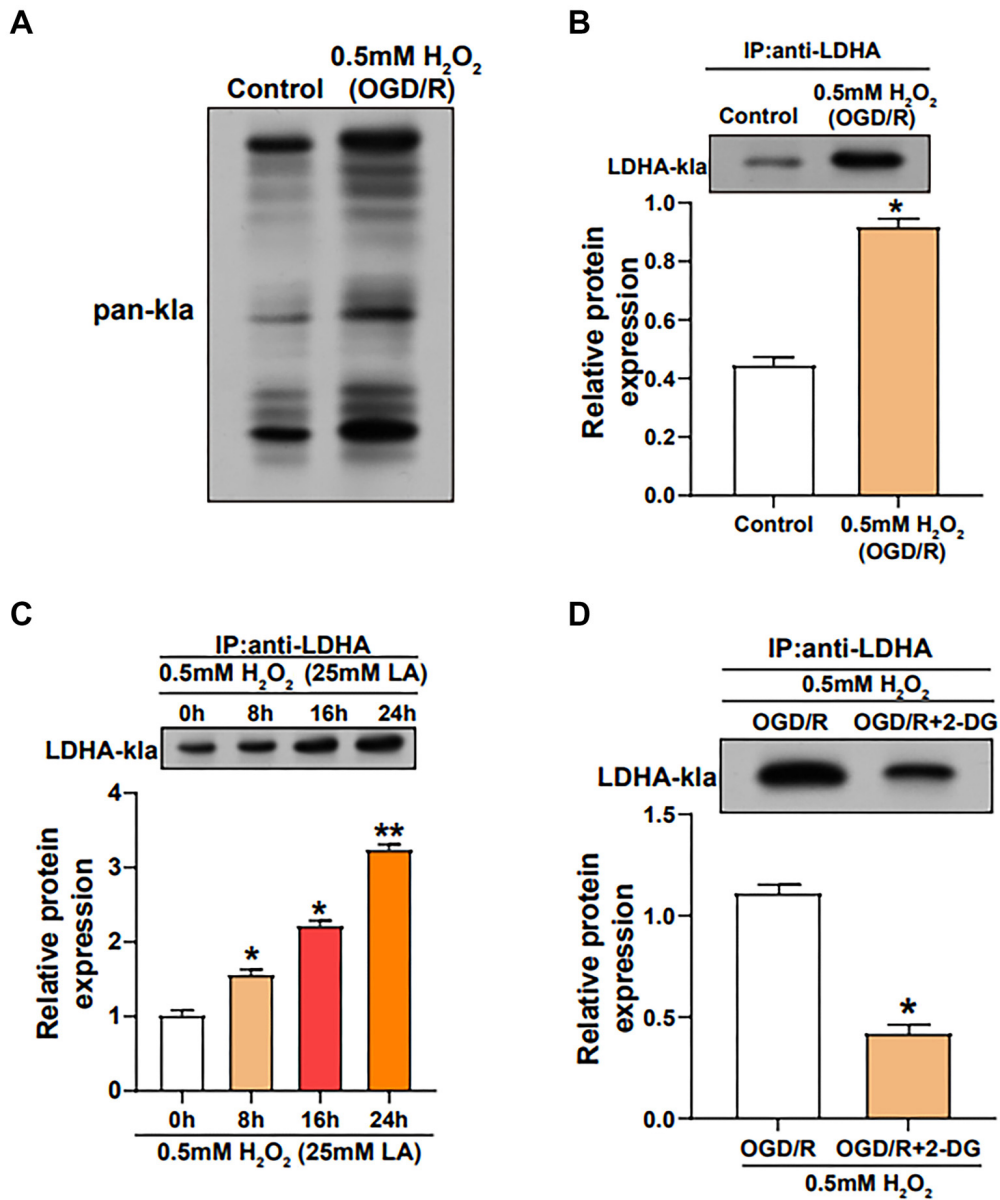
**Modulation of LDHA lactylation levels by OGD/R stimulation.** (A) IP and WB assays detecting the overall lactylation level of H_2_O_2_-treated VECs stimulated by OGD/R; (B) The lactylation level of LDHA in H_2_O_2_-treated VECs stimulated by OGD/R, detected by IP and WB assay; (C) IP and WB assays performed to detect the lactylation level of LDHA in H_2_O_2_-treated VECs treated with 25 mM LA for different times; (D) IP and WB assays performed to detect the lactylation level of LDHA in H_2_O_2_-treated VECs stimulated by OGD/R and 2-DG. *P < 0.05; **P < 0.01. LDHA: Lactate dehydrogenase A; WB: Western blotting; OGD/R: Oxygen-glucose deprivation/reperfusion; LA: Lactic acid; 2-DG: 2-deoxyglucose; IP: Immunoprecipitation; VECs: Vascular endothelial cells.

The overall lactylation levels in VECs under OGD/R-induced oxidative stress were assessed using the IP and WB methods. The results indicated a significant enhancement of overall lactylation levels in VECs subjected to OGD/R stimulation (Figure 8A). Additionally, a significant increase in the lactylation level of LDHA was observed in cells under OGD/R stress (Figure 8B). After LA treatment, the lactylation level of LDHA in cells increased in a dose-dependent manner (Figure 8C). Interestingly, treatment with 2-DG significantly down-regulated the lactylation level of LDHA in OGD/R-stressed VECs (Figure 8D).

These findings reveal significant lactate-related genes and their functional enrichment in IA tissues compared to normal tissues. Through differential expression analysis, we identified 63 overlapping genes that are enriched in metabolic processes, cellular components, and molecular functions related to glycolysis and lactate metabolism. PPI network analysis highlighted seven key genes (*ENO2*, *HK1*, *MDH2*, *ALDOB*, *LDHA*, *PKLR*, and *TALDO1*) with notable expression differences between IA and normal tissues. Among these, *LDHA* emerged as a hub gene critical for lactate production and cellular energy metabolism. Further experiments revealed that *LDHA* modulation impacts VEC proliferation and migration under oxidative stress conditions. Specifically, *LDHA* overexpression conferred protective effects against oxidative damage, enhancing cell survival and reducing apoptosis, while *VEGFA* knockdown negated these benefits. Additionally, OGD/R-induced oxidative stress led to altered *LDHA* expression, increased glycolysis, and lactate production in VECs. The modulation of lactate levels affected cell viability and apoptosis, with excess lactate exacerbating oxidative damage. Finally, *LDHA* lactylation levels were significantly regulated under stress conditions, highlighting the complex role of *LDHA* in cellular stress responses. These findings underscore the pivotal role of lactate metabolism and *LDHA* in the pathophysiology of IA and suggest potential therapeutic targets for mitigating oxidative damage in VECs.

## Discussion

CA, arising from abnormal swelling of cerebral blood vessels, pose a severe threat to life upon rupture [40]. The release of inflammatory mediators stimulates the production of large amounts of inflammatory cytokines and oxidative factors, leading to remodeling of phenotypic regulation and dysfunction in the ECM of VSMCs. Inflammatory responses, oxidative damage, and cell death are closely related to the etiology of CA [41]. Under normal physiological conditions, a dynamic balance exists between the proliferation and apoptosis of VSMCs. Endothelial dysfunction and phenotypic transformation of VSMCs contribute significantly to aneurysm development [42]. H_2_O_2_ is an oxidative stress inducer capable of causing apoptosis in VSMCs. H_2_O_2_ has been used to establish an in vitro aneurysm model by inducing apoptosis in VSMC cells [43], providing a useful tool for studying the underlying mechanisms and potential treatments for aneurysms. Insights from Tawk et al. [44] highlight a shift from size-based approaches to comprehensive risk assessment for unruptured intracranial aneurysms (UIA) and aneurysmal SAH treatment, emphasizing the preferential use of endovascular coil embolization in SAH therapy to improve outcomes. Texakalidis et al. [45] provided a comprehensive overview of the pathobiology of CA, detailing inflammatory pathways, genetics, and risk factors influencing their formation, growth, and rupture, thus offering a holistic perspective on the biological and physical aspects underlying CA development. Additionally, Xu et al. [46] explored the effects of glycolysis and lactate-related lactylation on lipid deposition, calcification, and angiogenesis in atherosclerosis, highlighting their potential as targeted intervention pathways. In our study, functional enrichment analysis of lactate-related genes in the CA context revealed significant involvement in glycolytic processes, pyruvate metabolism, lysosomal cavity, mitochondrial matrix, LDH activity, oxidoreductase activity, glycolysis/gluconeogenesis, fructose and mannose metabolism, and pyruvate metabolism. Our enrichment analysis results underscored a dependence on glycolysis, consistent with previous studies’ findings. Elevated glycolytic activity leads to the accumulation of pyruvate and its conversion to lactate in the absence of adequate OXPHOS, potentially creating an acidic microenvironment that may affect the stability and progression of CA. Additionally, genes related to lysosomal and mitochondrial processes were highly enriched, suggesting that changes in organelle function may impact energy metabolism and cellular homeostasis, ultimately leading to vessel wall thinning and the development of aneurysms.

Lactylation modification, involving the acylation of lysine residues by lactate molecules, plays a pivotal role in regulating cellular metabolism [47, 48]. Building on substantial research on lactylation, Zhang et al. [49] discovered the dynamic regulation of lactylation on lysine 1897 of α-myosin heavy chain (α-MHC), influencing interactions with muscle-associated proteins and impacting cardiac structure and function. The reduced lactylation of α-MHC at K1897 in heart failure is attributed to diminished intracellular lactate levels. There is potential for mitigating heart failure by modulating this sarcomeric interaction through the control of lactate concentration. Furthermore, Fan et al. [50] demonstrated that elevated lactate levels post-myocardial infarction induce Snail1 lactylation via the TGF-β/Smad2 pathway, promoting endothelial-to-mesenchymal transition (EndoMT), thereby exacerbating cardiac fibrosis and functional impairment, unveiling a previously unrecognized role of lactate in exacerbating adverse cardiac outcomes. In gliomas, LDHA expression is significantly elevated and positively correlated with M2 type tumor-associated macrophage (TAM) infiltration, where LA secreted by glioma cells induces TAM polarization to the M2 subtype, subsequently promoting glioma cell proliferation, migration, invasion, and mesenchymal transformation [51]. Circular RNA derived from the mitogen-activated protein kinase 9 (Circ_MAPK9) influences hepatocellular carcinoma progression through silencing miR-642b-3p, thereby promoting the STAT3 and LDHA expression [52]. In our study, bioinformatics analysis of the GSE26969 dataset revealed that *LDHA*, a hub gene involved in lactate metabolism, was significantly downregulated in IA samples. Functional analysis indicated that *LDHA* regulation significantly affects VEC proliferation and migration under oxidative stress, suggesting its potential role in the pathogenesis of CA.

VEGFA, a member of the platelet-derived growth factor (PDGF)/VEGF growth factor family, has been found to induce the proliferation and migration of VECs, playing a crucial role in physiological and pathological angiogenesis [53, 54]. Lactate, potentially by influencing transcription factors, signaling pathway molecules, or other regulatory factors, modulates VEGFA expression levels either directly or indirectly [55]. Dong et al. [56] observed the elevated expression of hypoxia-inducible factor-1 alpha (HIF-1α) and its target genes, *VEGFA* and *LDHA*, in Parkinson’s disease (PD) upon exposure to MPP(+), accompanied by suppressed expression of sirtuin 1 (*SIRT1*), suggesting a potential link between PD pathophysiology and the dysregulation of the SIRT1/HIF-1α signaling axis. Additionally, Liu et al. found a correlation between the progression of CA and a decrease in SMCs and ECs within the aneurysm wall, along with an increase in MMPs and a decrease in collagen levels, linked to reduced *VEGFA* expression in ECs. These findings highlight potential therapeutic targets for preventing CA progression [57]. Chédeville et al. [58] revealed that hypoxia-induced upregulation of *LDHA* in glioblastoma triggers a glycolytic shift accompanied by enhanced expression of pro-angiogenic factors, including *VEGFA*, with high expression levels of genes, such as *SLC2A1*, *LDHA*, *PDK1*, *PFKFB4*, *HK2*, *VEGFA*, *SERPINE1*, *TFRC*, and *ADM* significantly associated with decreased survival rates. These results underscore the potential of these factors as effective therapeutic targets for glioblastoma. Our experimental analysis demonstrates that co-introduction of *VEGFA* into cells overexpressing *LDHA* rescues the initially observed increase in proliferation and reduction in apoptosis, implying an interaction between *VEGFA* and *LDHA* in VECs in regulating cellular responses to oxidative stress.

At the onset of CA, local blood circulation is compromised, leading to ischemic-hypoxic injury in the affected tissue [59]. Building on the findings of Yao et al. [27], who demonstrated the crucial role of *LDHA* in CI/R injury, our study delved into the complex molecular landscape associated with *LDHA* in CA progression. Investigating the impact of OGD/R stimulation, Pan et al. [60] identified an upregulation of monocarboxylate transporter 4 (MCT4), which facilitates a glycolytic shift by regulating LDHA and suppressing OXPHOS, contributing to cardiac protection in myocardial I/R injury. Furthermore, Lu et al. [61] revealed that reoxygenation-induced VEC injury led to a significant reduction in nuclear receptor subfamily 4 group a member 3 (NR4A3) protein levels, with the antioxidant steroid TRIOL countering this effect by inhibiting ROS-driven ubiquitination and degradation, highlighting a novel post-translational regulation involving NR4A3 and the swi/snf-related, matrix-associated, actin-dependent regulator of chromatin, subfamily b, member 1 (SMARCB1) interactions in VECs. In our investigation, we observed a significant increase in *LDHA* lactylation in VECs under OGD/R-induced oxidative stress, which was further augmented by LA treatment, suggesting a dose-dependent effect. While lactate accumulation exacerbates oxidative damage, *LDHA* overexpression enhances VEC recovery. Interestingly, 2-DG inhibits glycolysis, reducing cell death and linking glycolytic activity to oxidative stress responses. Under 2-DG regulation, *LDHA* lactylation increased under stress, indicating a regulatory role for post-translational modifications in cellular responses to oxidative stress.

Our study highlights the significant role of lactate metabolism and *LDHA* regulation in the pathogenesis of CA. The findings suggest that targeting metabolic pathways, particularly glycolysis and lactate-related processes, could offer new therapeutic strategies for CA. The interplay between *LDHA* and *VEGFA* in VECs under oxidative stress underscores the complexity of cellular responses in CA progression. Future research should continue exploring these molecular mechanisms, emphasizing developing targeted interventions to mitigate oxidative damage and stabilize vascular structures, potentially improving clinical outcomes for patients with CA. The strengths of this study lie in its comprehensive bioinformatics and functional analysis, providing a robust foundation for future investigations. However, the study’s limitations include the need for validation in larger, diverse cohorts and the exploration of additional molecular pathways involved in CA development. The implications of this research extend beyond CA, offering insights into the broader field of vascular biology and disease.

## Conclusion

In summary, our comprehensive analysis delved into the multifaceted role of *LDHA* in CA. Particularly, under oxidative stress induced by OGD/R, *LDHA* expression exhibited significant regulation, profoundly influencing cellular metabolic processes. We observed a noteworthy correlation wherein *LDHA* downregulation led to increased glycolytic flux, evidenced by heightened glucose uptake and lactate production, suggesting a compensatory response by VECs to oxidative stress. Additionally, *LDHA* overexpression exhibited a protective role against oxidative stress-induced damage, promoting cell viability and reducing apoptosis. Notably, the protective effect of *LDHA* overexpression was reversed by the downregulation of *VEGFA*. Additionally, we identified lactylation of *LDHA* as a pivotal post-translational modification responsive to metabolic shifts under stress conditions. This modification appears to serve as a regulatory mechanism, facilitating VECs’ adaptation to oxidative stress induced by OGD/R. These findings elucidate the role of lactate metabolism and *LDHA* regulation, enhancing our understanding of cellular adaptations in CA. They also highlight potential therapeutic targets for mitigating disease progression in the future.

## Data Availability

The datasets used and/or analyzed during the current study are available from the corresponding author upon reasonable request.
